# Comparative analysis of the quality of execution of road surfaces on newly built, reconstructed and renovated roads in the city Płock area (Poland)

**DOI:** 10.1038/s41598-024-55707-0

**Published:** 2024-03-01

**Authors:** Piotr Gryszpanowicz, Natalia Gasik-Kowalska, Konrad J. Waluś

**Affiliations:** 1 Warsaw University of Technology, Warsaw, Poland; 2 Poznan University of Technology, Poznan, Poland

**Keywords:** Road construction, Durability of the road surface, Acceptance of road surfaces, Road condition monitoring, Road safety, Runtime errors, Civil engineering, Energy infrastructure, Mechanical engineering

## Abstract

Carrying out repair works, reconstruction, and construction of new road surfaces is a permanent element of urban space. The quality of the new pavement for the adopted traffic category directly impacts the road infrastructure's durability. The choice of road surface structure depends on the adopted traffic category. The aim of the article is to assess the works carried out on selected road surfaces within the city of Płock (Poland) in terms of the technical specification requirements and the durability of road infrastructure. The paper presents the tests of three road layers: base layer, binding layer and wearing course. The tests were carried out on 11 streets, and 29 samples were collected.

## Introduction

The road infrastructure in urban areas is both extensive and diversified. There are surfaces made of various materials: concrete, paving stones, asphalt and others. Each of these surfaces is characterised by different characteristics of the coefficient of adhesion^1,2^, the anti-slip level and the rate and type of operational wear^3^. Therefore, renovation, reconstruction and construction of new road surfaces are a permanent element of activities in urban areas.

The variety of road infrastructure depends, among other things, on the intended use of a given road and its technical class. Additional elements may be traffic slowers and tram tracks^4,5^. Quantification of these factors directly affects the scope of road safety. Therefore, extensive research is carried out on types and kinds of surfaces, from typical road surfaces to special and airport surfaces. One of the most important aspects of the operation of road surfaces is the coefficient of adhesion or slipperiness obtained on them^6–8^. Another unfavorable phenomenon on roads for motor vehicles is the problem of aquaplaning^9–11^. Research related to the drainage of road surfaces is presented in Ref.^12^. A factor affecting the comfort and safety of vehicle traffic is the forecasting of the longitudinal evenness of the road surface depending on the change in the structure’s durability and wear and tear^13^. The characteristics of modern cement concrete surfaces and the description of the technologies and materials used for their repair are presented in Ref.^14^. Road tests are usually performed on surfaces in good condition, however, each road infrastructure is subject to aging processes, which makes it necessary to conduct ongoing monitoring of the surface condition^15,16^. Constant supervision supports decision-making processes related to the planning of repairs and the development of the road network^17,18^.

Productivity is a key function in all areas of infrastructure development, taking into account costs and time^19^. In road construction, efficiency is a key element in assessing whether the surface will function properly under traffic loads and environmental factors^20^. The surface deteriorates over time and the rate of deterioration varies greatly depending on the factors listed and the amount of maintenance work carried out by the road administration during the life of the surface. One of the methods of predicting potential road damage may be the analysis of the durability of mineral-asphalt mixtures for immersion in flood water^21^. The correct choice of materials and the right quality of construction will help slow down the rate of destruction^22^.

Planning road surface repairs is preceded by an assessment of the quality of the road surface and estimation of its capacity^23,24^. Supporting road surface maintenance decisions in terms of the adopted safety level has a significant impact on repair planning processes^25^. The identification of critical road sections that require immediate attention may suggest a decision matrix that takes into account, among other things, the impact of the degree of degradation on the history of road accidents and infrastructure safety^26^. An additional aspect of carrying out repair works is the estimation of the harmfulness of the renovation for the surrounding infrastructure and the environment^27–29^. An important environmental factor is also the use of polymer waste obtained from used plastic bottles or rubber crumbs from used car tires^30^. Research on the impact of road surface construction materials on the level of particulate emissions on the example of roads in Poland is presented in Ref.^31^. Another element of the documentation is the estimation of the amount of superstructure materials needed to repair the road^32^. Taking into account the conditions of renovation allows spatial planning of works and the time of their implementation^33–35^, as well as the preparation of documentation required by law along with technical specifications.

Road infrastructure requires periodic maintenance and repairs. There are special requirements for the roadway that must be followed during construction or repair. The uncertainty of composition, sensitivity to temperature and viscoelastic properties of road materials make the structural analysis of the surface difficult to perform^36^. Therefore, in addition to monitoring road surfaces, both invasive and non-invasive tests are performed. An example of such research is the analysis of mechanistic, volumetric, surface properties of road surfaces and sound absorption^37–39^. Regardless of the planned renovation works, metrological measurements are carried out to facilitate repairs in order to define the road geometry in detail^40^.

In order to support the development of road traffic, it is necessary to intensify research on road construction technology, constantly modernize construction technology and techniques, and then effectively ensure the rapid development of urban traffic. This will enable the development of urban transport and the quality of urban road projects^41^. Research on methods of improving the durability of road surfaces and the impact of the composition of asphalt mixtures on operating processes are also intensified^42–44^.

Project management at the renovation stage^45^, examination of road construction conditions^46^ and evaluation of these works after their completion is one of the most important conditions determining the authorisation of a road for traffic and the assessment of compliance with the technical specifications.

The aim of the article is to assess the works carried out on selected road surfaces within the city of Płock (Poland) in terms of the requirements of technical specification and durability of road infrastructure.

## Regulations on the construction, reconstruction and acceptance of roads in Poland

In Poland, regulations, requirements and guidelines for the design, subsequent implementation and acceptance of roads are contained in legal acts such as:

- Construction Law, which specifies the obligations and rights of participants in the entire construction process and administrative authorities. In addition, it defines the rules of criminal liability related to non-compliance with building regulations. The Construction Law is the basic document regulating activities related to the design, construction, maintenance and demolition of buildings.
- Act of 21 March 1985 on public roads. (Journal of Laws of 1985, No. 14, item 60)—defines all concepts related to objects intended for movement. The Act divides public roads into categories depending on, inter alia, their function in the road network.
- Regulation of the Minister of Transport and Maritime Economy of 2 March 1999 on technical conditions to be met by public roads and their location. (Journal of Laws of 1999, No. 43, item 430), and from 21.09.2022. Regulation of the Minister of Infrastructure of 24 June 2022 on technical and construction regulations for public roads (Journal of Laws of 2022, item 1518), which contains provisions specifying the technical conditions to be met by public roads, their location and use. The Ordinance includes road classes and corresponding speeds for design, lane width, road inclination. Requirements for parts of roads intended for bicycle traffic, public transport, parking lots, exits, intersections, etc. have been taken into account. In addition, fire regulations and requirements for underground installations and structures are included.
- Catalogue of typical structures of susceptible and semi-rigid surfaces—Annex to the Ordinance No. 31 of the General Directorate for National Roads and Motorways of 16.06.2014 defining typical surface structures based on fatigue criteria. This document provides instructions for the design of road infrastructure. The catalogue takes into account the influence of soil and water conditions, design movement and the construction of surface layers.
- Technical specification of execution and acceptance of construction works prepared by the designer—strictly defined technical requirements that should be met by a newly created or reconstructed road. The specification contains all the information necessary to make the surface in accordance with the project.

Acts and regulations are frequently updated due to the constant specification of legal acts. In addition, in recent years, there has been a clear tendency to implement the idea of sustainable development in road engineering, which results in supplementing the regulations with requirements for recycled materials, limiting the consumption of natural crushed aggregates, transported over long distances. In addition, efforts to increase the durability of road structures result in the creation of new technologies conducive to the development of road engineering.

## Methodology and results of research to assess the quality of works carried out on the streets of Płock

In the Polish area, guidelines for the surface of asphalt roads are defined both in standards and regulations of the construction law. The PN-EN 13108-1^47^ standard specifies the requirements to be met by asphalt products used in road engineering. In addition, the General Directorate for National Roads and Motorways has developed a number of design recommendations regarding the thickness and composition of individual layers of the surface. Usually, however, the quality of work is determined by the compliance of works with the provisions of the technical specification of the project, which is prepared individually for each road investment.

In order to avoid irregularities resulting from executive errors occurring during the implementation of works on road investments, researches are commissioned to determine the level of compliance of works with the requirements of the technical specification of the project. These researches include:

- analysis of the composition of the mineral-asphalt mixture determined on MMA samples taken during the incorporation of the mixture;
- testing of density, bulk density and content of free spaces in the mineral-asphalt mixture;
- layer compaction indicator;
- the content of free spaces in the layer;
- layer thickness;
- additionally, longitudinal and transverse evenness tests are carried out for the surface layer.

The analysis of the composition of the mineral-asphalt mixture consists in determining the content of soluble binder and determining the grain size of MMA (mineral-asphalt mixture). The binder content is determined in accordance with PN-EN 12697-1^48^, where the weighed analytical sample is placed in the extractor until the binder remains on the surface of the aggregate grains. After separation of the mineral parts from the extraction solution by means of, for example, a continuous centrifuge, the binder content is determined by a method based on the difference in mass or recovery (binder from the extraction solution). The particle size composition is determined in accordance with PN-EN 12697-2^49^. The test is conducted on material recovered from the determination of binder content in MMA. After drying to a constant weight and complete separation of the grains, the aggregate is subjected to sieve analysis, consisting of sieving through sieves of different mesh sizes, weighing each fraction and determining the percentage of mass of a given fraction in the total weight of the sample.

The density of the mineral-asphalt mixture is carried out on the basis of PN-EN 12697-5^50^. This standard presents three methods used in the determination of MMA density—the volumetric method (using a pycnometer), the hydrostatic method (water bath of the sample container) and the mathematical method, where it is necessary to know the composition of the mixture. The bulk density is determined on the basis of PN-EN 12697-6^51^, one of four methods—the method of bulk density in the dry state, in the saturated surface-dry state, in the surface seal state (protecting free spaces against water penetration, e.g. by paraffin) and on the basis of geometric dimensions (based on the mass and dimensions of the dry sample). The determination of the content of free spaces in MMA (according to PN-EN 12697-8^52^ is determined computationally using the density of the mineral-asphalt mixture and the bulk density MMA.

The determination of the layer compaction index in accordance with PN-EN 13108-20^53^ consists in calculating the share of the bulk density of the layer in relation to the reference density (after re-compacting the crushed core samples). The content of free spaces in the layer is determined analogously to the content of free spaces in the mineral-asphalt mixture. The layer thickness is tested in accordance with PN-EN 12697-36^54^ and consists in measuring each layer of the surface with an accuracy of 1 mm. The method of destructive measurement and electromagnetic measurement shall be used. In the destructive measurement method, the preparation of samples for determination consists in marking the core with boundary lines perpendicular to the upper and lower planes, and the layer thickness is determined as the arithmetic mean of the four measurements (every 90°). Electromagnetic measurement is carried out using a calibrated electromagnetic apparatus, where with the help of a counterpole attached to the road it is possible to measure the thickness of the layers without the need to take a core sample.

Equality tests carried out according to the recommendations of standard BN-68/8931-04^55^ consist in measuring equality using a four-meter patch or planograph. In the case of a planography—in the central part of the trolley (with a spacing of extreme wheels of 400 cm) there is a recording device and an indicator of unevenness, transferring the measurement results to a paper tape on a scale of 1:2. The pointer of the unevenness measuring device indicates the clearances in which the trolley measuring wheel is located. Measurement of unevenness with a patch consists in measuring the clearance between the lower edge of the patch and the surface layer in selected places using a scaled wedge.

Longitudinal evenness can also be measured using a measuring trailer^56^ or a laser profilograph; this method allows the determination of IRI (International Roughness Index), expressed in mm/m or m/km. The parameter characterizes the operation of the suspension in a conventionally adopted computational model of a vehicle moving at a constant speed of 80 km/h along a previously determined distance (usually—50 m). In accordance with the PN-EN 13036-6^57^ standard, the polygraph equipped with measurement sensors is mounted on a measurement vehicle. After completing the measurements, a report is generated on the monitor inside the vehicle, enabling the assessment of cross-section evenness and the determination of the IRI index.

Table 1 shows the cross-sections of typical road surfaces depending on the traffic category and its load. When starting the surface design process, it should be assumed what design movement may occur during the use of the structure. The concept of design traffic is understood as the total number of equivalent standard axes with a load of 100 kN, falling on the most loaded lane during the design period (in the case of analyzed streets it is—20 years). The equivalent standard axle is a design-approved replacement single axle with a single wheel and a load of 100 kN. It is used to convert the actual number of vehicles per lane. On the basis of the design movement, the traffic category (from KR1 to KR7, in polish KR—kategoria ruchu = traffic category) is determined in the form of ranges of values expressed in millions of equivalent axes.

**Table 1:**
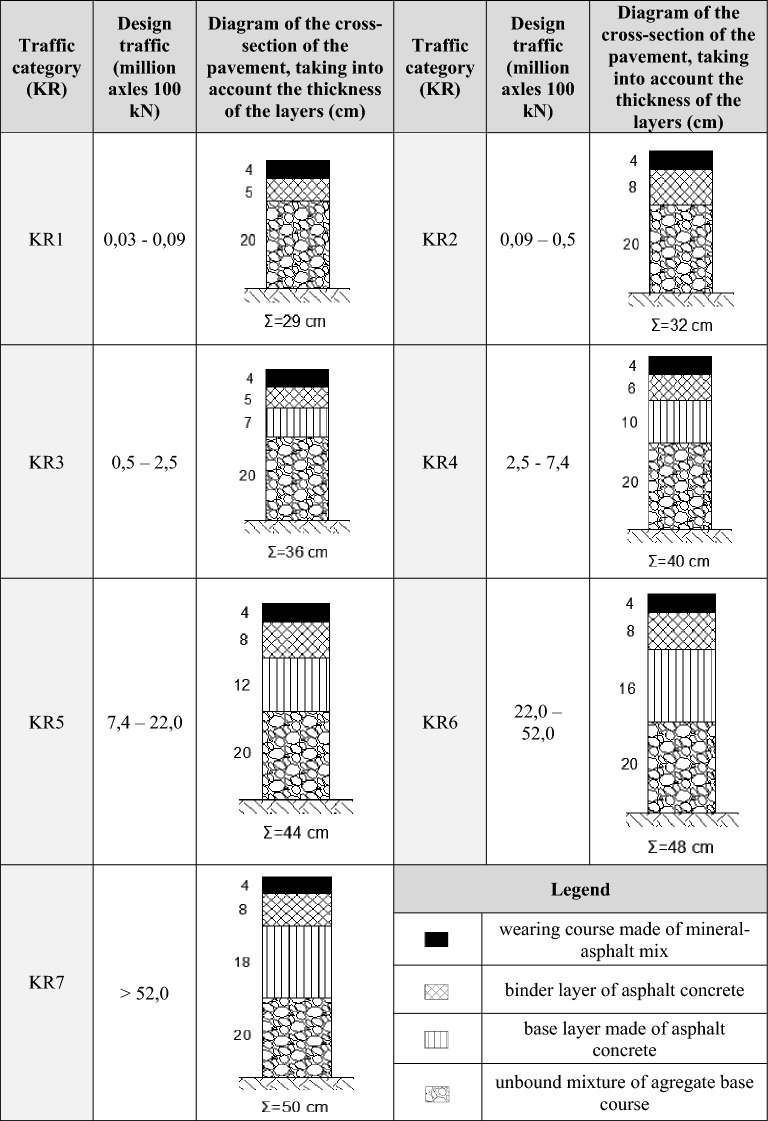
Typical constructions of the upper layers of flexible pavements. Source: own study based on the instructions of GDDKiA (in polish Generalna Dyrekcja Dróg Krajowych i Autostrad—General Directorate for National Roads and Motorways).

The research was carried out for three road layers—the foundation layer, which is the function of a ground stabilizer, binding—acting as a connector between the foundation layer and the wear layer, and the abrasion layer—the top layer of the surface. Drilling was carried out on 11 streets. The samples taken were used to verify the compliance of the work with the technical specification of the project. The tests were carried out in accordance with the guidelines contained in the relevant standards.

Each of the streets on which samples were taken for testing was designed in accordance with the traffic category adopted at the stage of assumptions (Table 2).

**Table 2:** List of the tested streets and traffic categories assigned at the design stage.

No.	Analyzed street	Designated traffic category
1	Kolejowa	KR5
2	Otolińska	KR4
3	Dobrzykowska	KR4
4	Grabówka	KR4
5	Przemysłowa	KR5
6	Kostrogaj	KR5
7	Wiadukt	KR5
8	Polna	KR4
9	Raczkowizna	KR4
10	Tysiąclecia	KR4
11	Zielona	KR4

**Figure 1:**
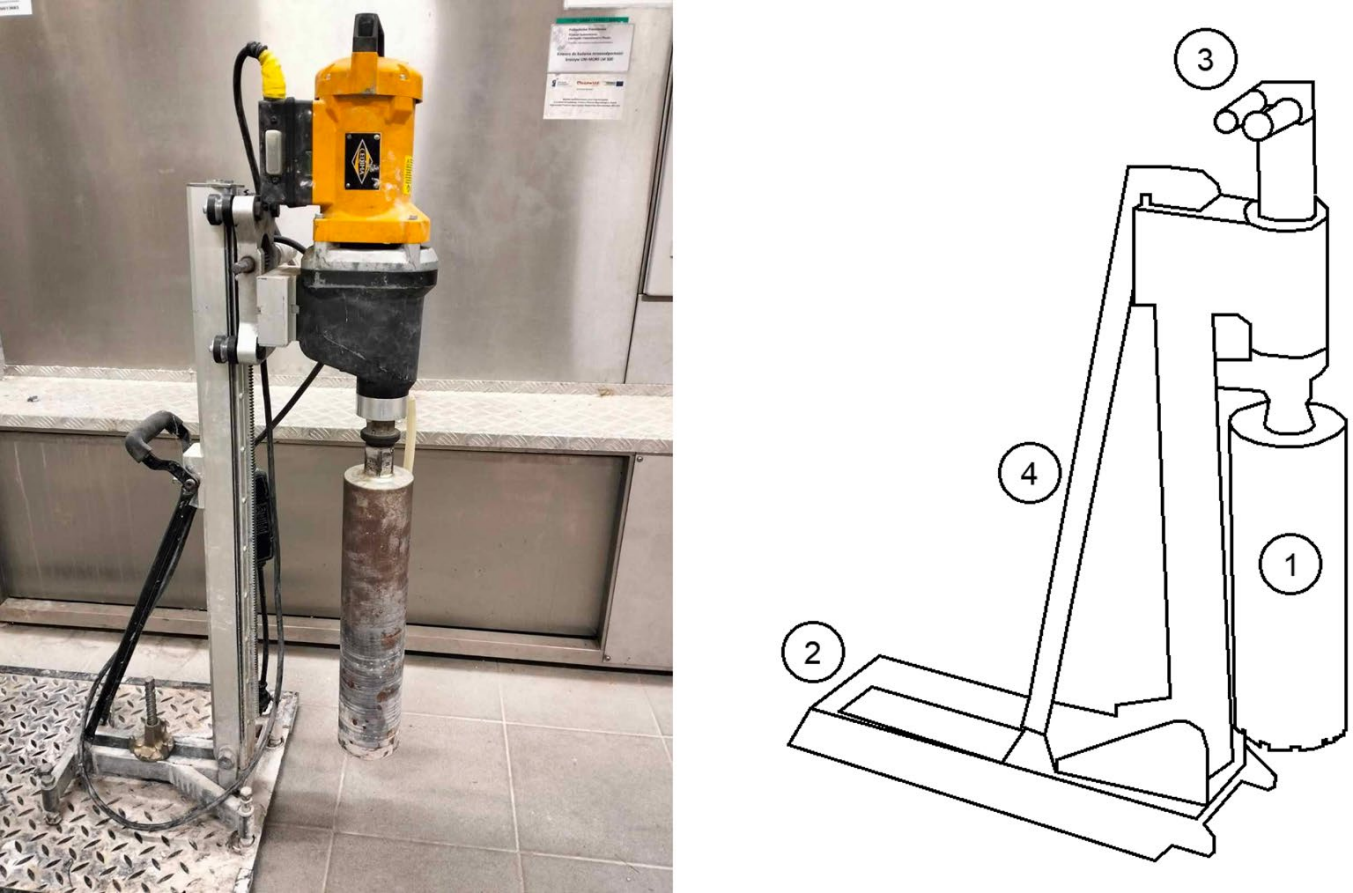
View and diagram of a hydraulic drilling rig used to collect samples (1—crown drill bit with a diameter of 100 mm; 2—ramp plate; 3—hydraulic power sockets, 4—bracket. (Own elaboration).

**Figure 2:**
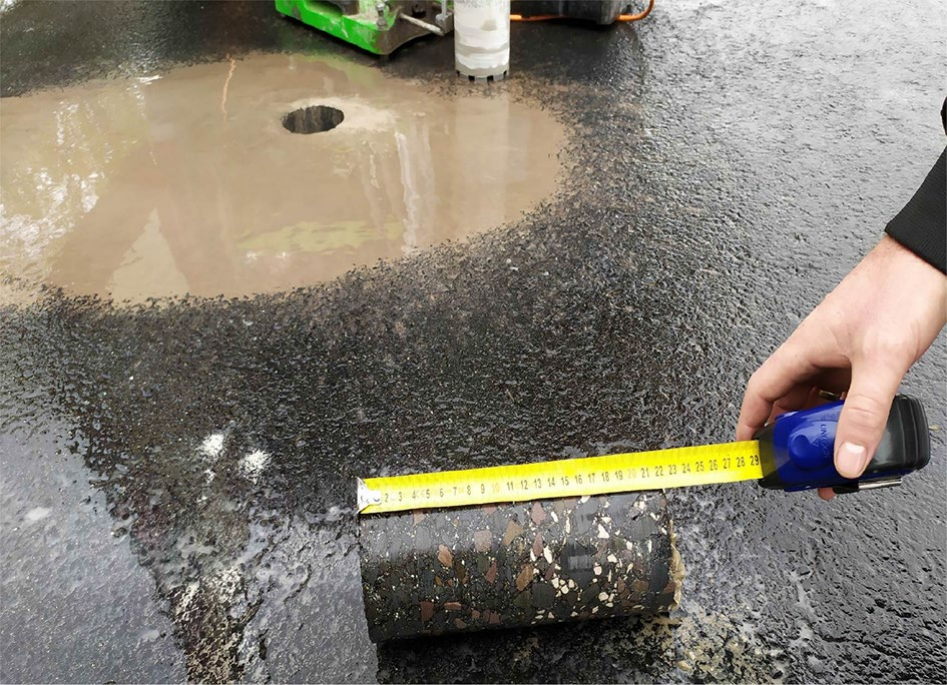
Core sample taken with the use of a hydraulic drilling rig.

**Figure 3:**
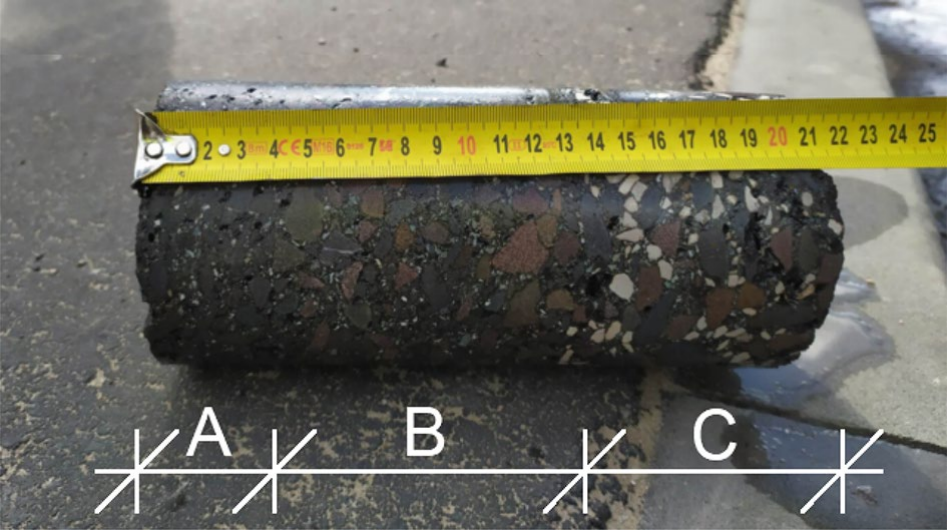
A core sample after taking it with a hydraulic drilling rig with a clear boundary between the individual layers. (**A**) Wearing course, (**B**) binding layer, (**C**) foundations.

The layer thickness was tested on core samples with a diameter of 100 mm. In accordance with PN-EN 12697-36^54^, the determination was carried out using the destructive measurement method using a hydraulic drilling rig (Fig. 1), where four measurements were evenly distributed around the perimeter of the core were made on each sample (Fig. 2). The thickness of each layer was defined as the average of the measurements with an accuracy of 1 mm (Fig. 3). The bulk density test of the mineral-asphalt mixture was carried out on the basis of PN-EN 12697-6^51^. The SSD (Saturated Surface Dry) method was chosen, i.e. the analysis of samples in the saturated state, where, after determining the dry mass, the samples were immersed in water until full soaking (constant mass of samples). The mass of the samples in water and in the saturated state after pre-drying was then determined. The compaction index was determined as the percentage ratio of bulk density and reference density, in accordance with the assumptions of PN-EN 13108-20 Annex C^53^. The content of free spaces in the layer was determined on the basis of PN-EN 12697-8^52^ using for this purpose the determined density of the mineral-asphalt mixture and the volumetric density MMA.

## Results of tests on the quality of works carried out on the streets of the city of Płock

**Figure 4:**
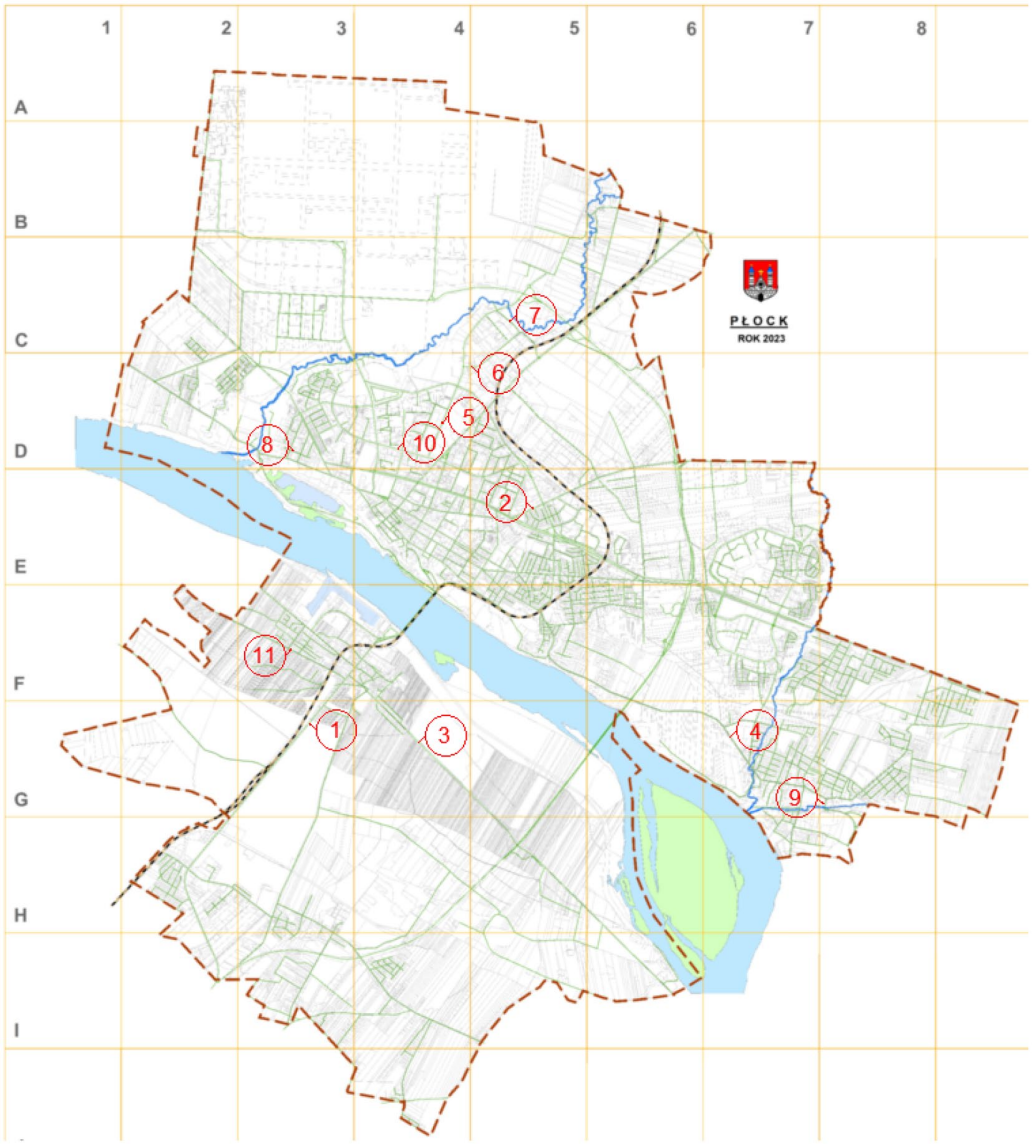
Map of the city of Płock with marked streets where samples for testing were taken. Legend: 1—street Kolejowa, 2—Otolińska, 3—Dobrzykowska, 4—Grabówka, 5—Przemysłowa, 6—Kostrogaj, 7—Wiadukt, 8—Polna, 9—Raczkowizna, 10—Tysiąclecia, 11—Zielona.

Sampling was carried out on the streets newly created, rebuilt and renovated, located within the city limits of Płock. Among the streets where boreholes were made, we distinguish: Kolejowa, Otolińska, Dobrzykowska, Grabówka, Przemysłowa, Kostrogaj, Viaduct, Polna, Raczkowizna, Tysiąclecia and Zielona streets (Fig. 4). A total of 29 core samples were taken. The number of wells necessary to be drilled was determined depending on the length of the section of the road investment being carried out. The research was carried out for each of the separated road layers and the purpose of the analysis is to check the compliance of the investment with the design assumptions. Irregularities arising between the conditions imposed by the relevant technical specifications and the quality of works have a direct impact on the durability of road infrastructure.

Table 3 summarises the results of research conducted for the foundation layer. Each of the projects was characterized by different design requirements. In the case of layer thickness tests, a difference in results of 10% of the value imposed by the technical specification (ST) was allowed. Bulk density was not included in the requirements of the construction documentation. The compaction index for each street should be greater than or equal to 98%. The percentage of free spaces in the layer has been determined by the technical specification as a range of permissible values.

**Table 3:** Test results of core samples and the requirements of the technical specification (ST) for each investment—foundation layer.

No.	Street name	Layer thickness in the sample (mm)	Bulk density MM-A in core sample (g/cm^3^)	Compaction index (%)	Free space in the layer (%)
Requirements		According to ST design	–	According to ST design	According to ST design
		140 ± 10%	–	≥ 98.0	1.0–4.5
1	Kolejowa	140	2479	100	1.9
		148	2493	100	1.4
Requirements		According to ST design	–	According to ST design	According to ST design
		100 ± 10%	–	≥ 98.0	4.0–7.0
2	Otolińska	99	2435	98	6.7
		115	2445	98	6.5
Requirements		According to ST design	–	According to ST design	According to ST design
		100 ± 10%	–	≥ 98.0	3.0–8.0
3	Dobrzykowska	93	2410	100	5.1
		94	2400	100	5.5
		101	2429	101	4.3
Requirements		According to ST design	–	According to ST design	According to ST design
		70 ± 10%	–	≥ 98.0	3.0–8.0
4	Grabówka	67	2441	100	4.3
		78	2416	100	5.5
Requirements		According to ST design	–	According to ST design	According to ST design
		100 ± 10%	–	≥ 98.0	4.5–8.0
5	Przemysłowa	101	2483	102	4.2
		118	2444	100	5.7
		95	2327	98	10
Requirements		According to ST design	–	According to ST design	According to ST design
		120 ± 10%	–	≥ 98.0	4.5–8.0
6	Kostrogaj	133	2404	100	6.3
		115	2424	101	5.5
		116	2468	102	3.8
		130	2465	102	3.9
Requirements		According to ST design	–	According to ST design	According to ST design
		120 ± 10%	–	≥ 98.0	4.5–8.0
7	Wiadukt	116	2462	101	5.3
Requirements		According to ST design	–	According to ST design	According to ST design
		80 ± 10%	–	≥ 98.0	4.5–8.0
8	Polna	74	2367	99	6.6
		74	2355	98	7.1
Requirements		According to ST design	–	According to ST design	According to ST design
		100 ± 10%	–	≥ 98.0	4.5–8.0
9	Raczkowizna	120	2465	99	6.1
		100	2513	100	5.9
		112	2561	101	2.8
Requirements		According to ST design	–	According to ST design	According to ST design
		100 ± 10%	–	≥ 98.0	3.0–8.0
10	Tysiąclecia	128	2500	102	3.0
		127	2476	101	4.0
		85	2372	98	7.9
		79	2391	98	9.0
Requirements		According to ST design	–	According to ST design	According to ST design
		70 ± 10%	–	≥ 98.0	3.0–8.0
11	Zielona	79	2417	101	5.3
		96	2447	102	4.1
		76	2428	102	4.8

In addition, the mean value, standard deviation and measurement uncertainty of the volumetric density of the mineral-asphalt mixture were calculated. The statistical analysis was based on the probability distribution of the Student's t-with a confidence interval of 95%.

The standard deviation (dispersion coefficient of results) is determined from the formula:

1 $$\sigma =\sqrt{\frac{1}{n-1}\sum_{i=1}^{n}{(x}_{i}-{\overline{x })}^{2}}$$

where: $\overline{x }$ is the arithmetic mean of the results obtained; ${x}_{i}$ is the single result value; $n$ is the number of results in the series. $\sigma$ is the standard deviation of a single measurement.

The coefficient of variation was determined from the formula:

2 $$\nu =\frac{\sigma }{\overline{x} }\cdot 100\%$$

where: $\nu$ is the coefficient of variation.

Measurement uncertainty was determined on the basis of the confidence interval of the Student's t-distribution according to the formula:

3 $$[\overline{x }-\frac{\sigma {u}_{\alpha }}{\sqrt{n-1}},\overline{x }+\frac{\sigma {u}_{\alpha }}{\sqrt{n-1}}]$$

where: ${u}_{\alpha }$ is the variable with t-Student distribution with n − 1 degrees of freedom.

For 29 results in the series—28 degrees of freedom: ${u}_{\alpha }=2048$.

The results of the statistical analysis are presented in Tables 4, 5 and 6.

**Table 4:** Results of statistical analysis of MM-A bulk density tests in the borehole for the foundation layer.

Bulk density MM-A in core sample			
Arithmetic mean density MMA $\overline{x }$ [g/cm^3^]	Standard deviation $\sigma$ [g/cm^3^]	Uncertainty of measurement (g/cm^3^)	Coefficient of variation $\nu$ [%]
2439	0.050	± 0.020	2.071

**Table 5:** Results of statistical analysis of MM-A bulk density tests in the wellbore for the binding layer.

Bulk density MM-A in core sample			
Arithmetic mean density MMA $\overline{x }$ (g/cm^3^)	Standard deviation $\sigma$ (g/cm^3^)	Uncertainty of measurement (g/cm^3^)	Coefficient of variation $\nu$ (%)
2471	0.100	± 0.037	3.856

**Table 6:** Results of statistical analysis of MM-A bulk density tests in the borehole for the wearing course.

Bulk density MM-A in core sample			
Arithmetic mean density MMA $\overline{x }$ (g/cm^3^)	Standard deviation $\sigma$ (g/cm^3^)	Uncertainty of measurement (g/cm^3^)	Coefficient of variation $\nu$ (%)
2458	0.112	± 0.043	4.54

Table 7 shows the results for the binding layer. Similarly as for the foundation layer, a difference of 10% in the results of thickness tests is allowed. The compaction index for each of the test samples should be more than or 98%. The percentage of free spaces is defined as a range of acceptable values.

**Table 7:** Test results of core samples and requirements of the technical specification (ST) for each investment—binding layer.

No.	Street name	Layer thickness in the sample (mm)	Bulk density MM-A in core sample (g/cm^3^)	Compaction index (%)	Free space in the layer (%)
Requirements		According to ST design	–	According to ST design	According to ST design
		90 ± 10%	–	≥ 98.0	1.0–4.5
1	Kolejowa	92	2416	99	3.0
		94	2455	100	1.1
Requirements		According to ST design	–	According to ST design	According to ST design
		60 ± 10%	–	≥ 98.0	4.0–7.0
2	Otolińska	58	2435	98	5.7
		65	2436	98	5.7
Requirements		According to ST design	–	According to ST design	According to ST design
		60 ± 10%	–	≥ 98.0	3.0–7.0
3	Dobrzykowska	54	2373	99	5.6
		51	2350	98	6.5
		60	2288	96	8.9
Requirements		According to ST design	–	According to ST design	According to ST design
		50 ± 10%	–	≥ 98.0	3.0–8.0
4	Grabówka	54	2413	99	4.9
		50	2415	100	5.2
Requirements		According to ST design	–	According to ST design	According to ST design
		90 ± 10%	–	≥ 98.0	4.5–8.0
5	Przemysłowa	83	2587	99	6.7
		98	2669	102	4.3
		66	2645	101	5.2
Requirements		According to ST design	–	According to ST design	According to ST design
		80 ± 10%	–	≥ 98.0	4.5–8.0
6	Kostrogaj	111	2487	100	4.2
		69	2432	98	6.3
		78	2643	102	3.3
		70	2647	102	3.2
Requirements		According to ST design	–	According to ST design	According to ST design
		80 ± 10%	–	≥ 98.0	4.5–8.0
7	Wiadukt	83	2486	101	4.0
Requirements		According to ST design	–	According to ST design	According to ST design
		60 ± 10%	–	≥ 98.0	4.5–8.0
8	Polna	45	2362	97	8.1
		63	2401	98	6.6
Requirements		According to ST design	–	According to ST design	According to ST design
		60 ± 10%	–	≥ 98.0	4.5–8.0
9	Raczkowizna	63	2508	101	2.9
		62	2386	97	8.0
		65	2514	102	2.5
Requirements		According to ST design	–	According to ST design	According to ST design
		60 ± 10%	–	≥ 98.0	3.0–7.0
10	Tysiąclecia	48	2479	100	3.5
		65	2513	103	1.8
		79	2507	101	5.1
		79	2519	98	4.9
Requirements		According to ST design	–	According to ST design	According to ST design
		60 ± 10%	–	≥ 98.0	3.0–8.0
11	Zielona	51	2413	99	5.7
		55	2475	101	3.3
		52	2419	99	5.5

For the binding layer, a higher value of standard deviation and measurement uncertainty of the results of bulk density tests relative to the foundation layer was obtained.

The results of the wear layer tests are summarised in Table 8. The requirements of the technical specifications were adopted in the same way as in the case of the two previously analyzed layers. The difference appeared in the design guidelines for the compaction index. Kolejowa Street is the only one of the considered surfaces that allows a value greater than or equal to 97% for the correct result of the compaction index, in other cases, it is 98%.

**Table 8:** Test results of core samples and technical specification requirements for each investment—wearing course.

No.	Street name	Layer thickness in the sample (mm)	Bulk density MM-A in core sample (g/cm^3^)	Compaction index (%)	Free space in the layer (%)
Requirements		According to ST design	–	According to ST design	According to ST design
		40 ± 10%	–	≥ 97.0	2.0–6.0
1	Kolejowa	42	2354	98	4.9
		45	2211	93	10.1
Requirements		According to ST design	–	According to ST design	According to ST design
		40 ± 10%	–	≥ 98.0	4.0–7.0
2	Otolińska	51	2506	98	4.4
		35	2478	97	5.0
Requirements		According to ST design	–	According to ST design	According to ST design
		40 ± 10%	–	≥ 98.0	2.0–5.0
3	Dobrzykowska	41	2312	99	4.6
		38	2334	100	3.7
		40	2325	100	4.1
Requirements		According to ST design	–	According to ST design	According to ST design
		40 ± 10%	–	≥ 98.0	1.5–5.0
4	Grabówka	45	2393	99	3.1
		38	2363	98	4.8
Requirements		According to ST design	–	According to ST design	According to ST design
		40 ± 10%	–	≥ 98.0	3.0–5.0
5	Przemysłowa	39	2601	100	1.8
		40	2633	99	2.3
		42	2617	100	1.2
Requirements		According to ST design	–	According to ST design	According to ST design
		40 ± 10%	–	≥ 98.0	3.0–5.0
6	Kostrogaj	42	2371	94	8.8
		46	2366	94	8.7
		42	2626	99	4.0
		39	2613	98	4.0
Requirements		According to ST design	–	According to ST design	According to ST design
		40 ± 10%	–	≥ 98.0	3.0–5.0
7	Wiadukt	36	2583	98	5.1
Requirements		According to ST design	–	According to ST design	According to ST design
		40 ± 10%	–	≥ 98.0	3.0–5.0
8	Polna	45	2373	96	6.3
		44	2368	96	6.5
Requirements		According to ST design	–	According to ST design	According to ST design
		40 ± 10%	–	≥ 98.0	3.0–5.0
9	Raczkowizna	42	2538	98	3.9
		38	2522	98	4.5
		41	2561	99	3.0
Requirements		According to ST design	–	According to ST design	According to ST design
		40 ± 10%	–	≥ 98.0	1.5–5.0
10	Tysiąclecia	36	2456	96	6.8
		42	2546	99	3.3
		45	2450	97	7.3
		43	2409	96	8.8
Requirements		According to ST design	–	According to ST design	According to ST design
		40 ± 10%	–	≥ 98.0	2.0–5.0
11	Zielona	51	2448	98	6.1
		40	2462	98	5.6
		39	2471	98	5.2

The results of the volume density tests of the wear layer samples indicate the greatest variation in values in relation to the other layers—the foundation and the binding.

## Analysis of the results of tests on the quality of works carried out on the streets of the city of Płock

The results of tests of the thickness of the foundation layer in the samples showed that 58.6% of the analyzed road foundations met the requirements of the technical specification. However, in 34.5% of the examined wells, the difference between the value adopted in the project and the result of the determination did not exceed 5%. 12 out of 29 samples do not meet the conditions of the technical documentation, and thus the investment in these places was not carried out in accordance with the project. Errors in the thickness of the foundation layer ranged from 1 mm on Grabówka and Kostrogaj streets to over 15 mm on Tysiąclecia and Zielona streets, which is a significant deviation from the technical specification guidelines.

**Figure 5:**
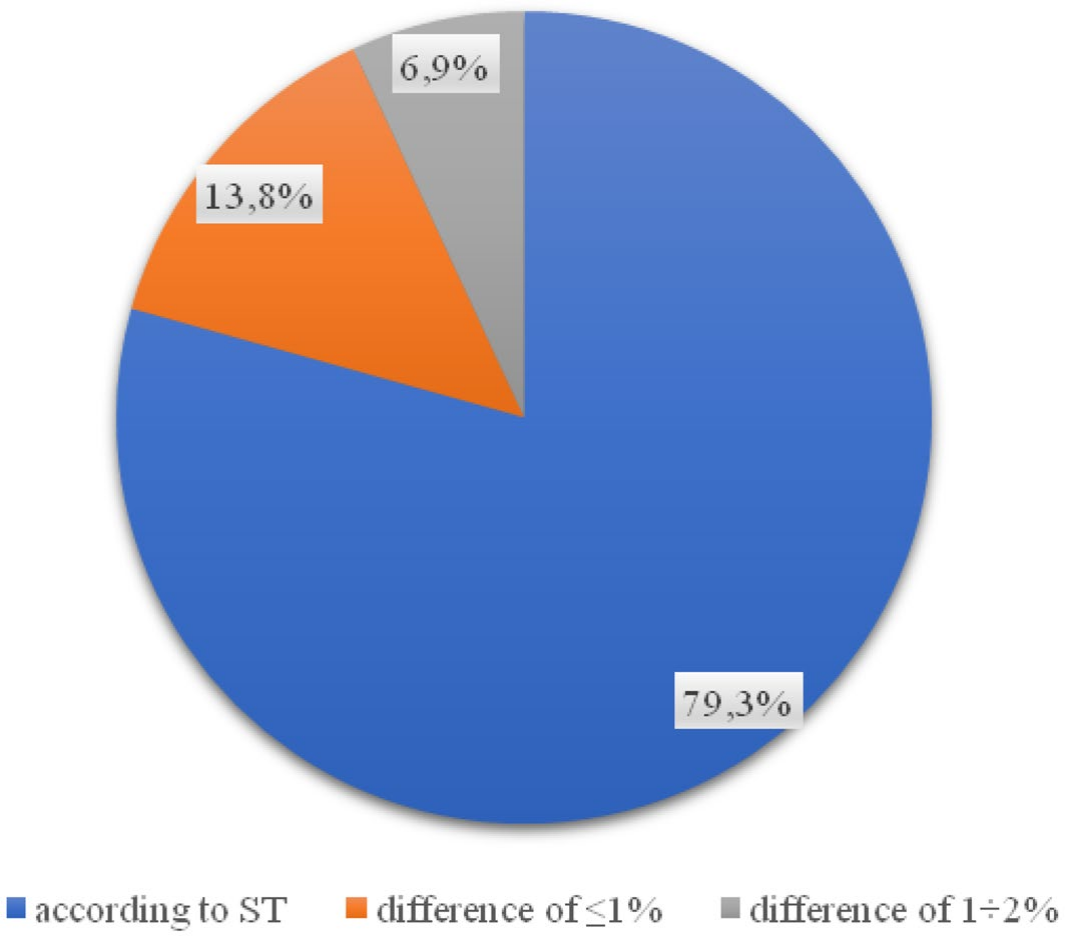
Percentage of values of free spaces in the foundation layer.

**Figure 6:**
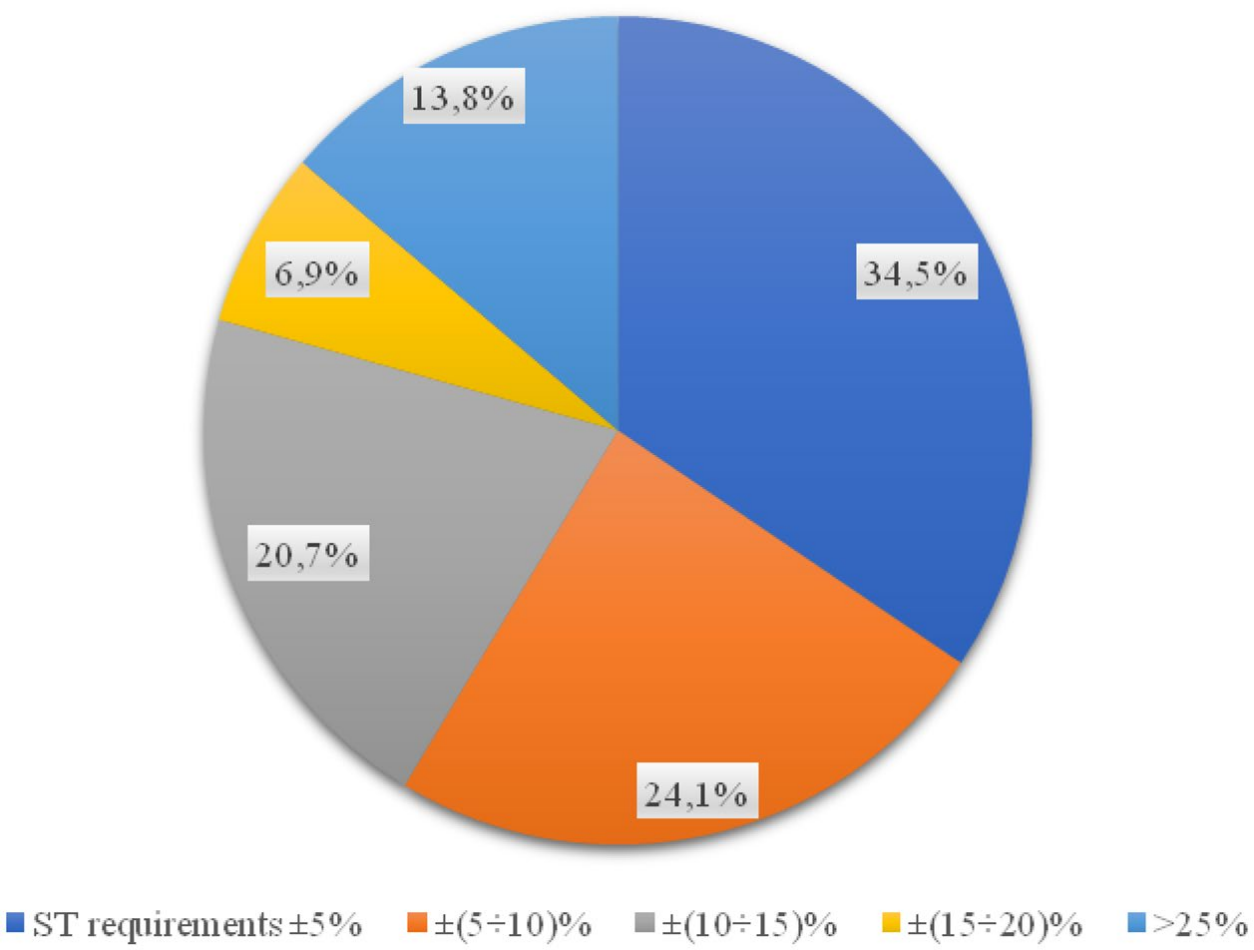
Percentage of base layer thickness in samples.

The value of the compaction index obtained in the tests for each of the 29 samples is in accordance with the requirements for it. In the case of the percentage of free space in the foundation layer, a significant part of the wells taken (23 out of 29) indicates the correct execution of the layer (Fig. 5). 20.7% of the analyzed samples do not comply with the technical specification (Fig. 6).

**Figure 7:**
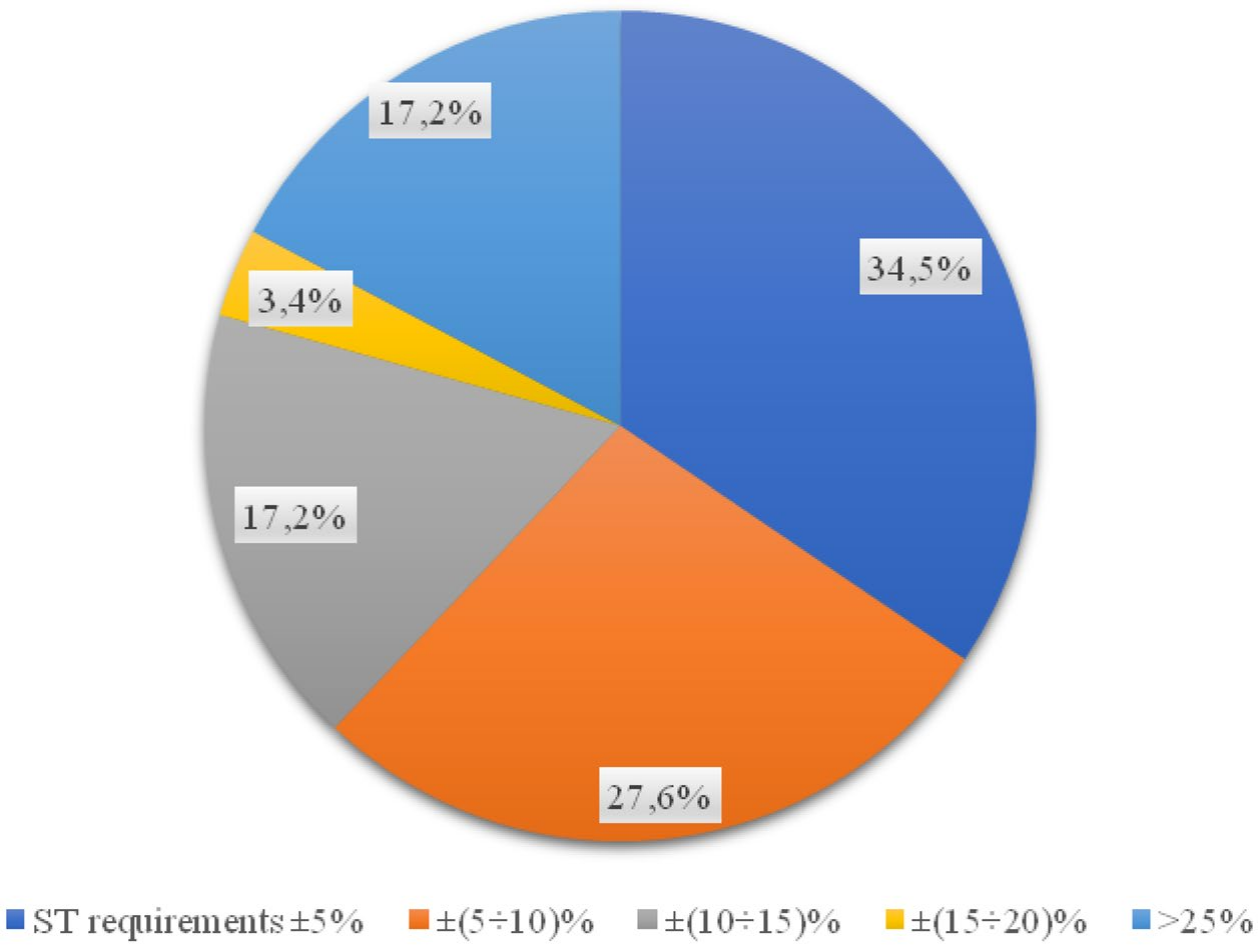
Percentage of the thickness of the binder layer in the samples.

For the bonding layer, the results of layer thickness tests showed that 62.1% of the core samples meet the design assumptions (Fig. 7). In 11 of the 29 wells drilled, the thickness of the binding layer does not comply with the guidelines contained in the relevant technical specifications of the project. Irregularities in the thickness of the binding layer ranged from 2 mm on Zielona Street to as much as 23 mm on Kostrogaj Street. Such implementation errors may generate social consequences, especially in terms of road safety, which may be related to faster wear of the road surface and the development of longitudinal and transverse unevenness.

**Figure 8:**
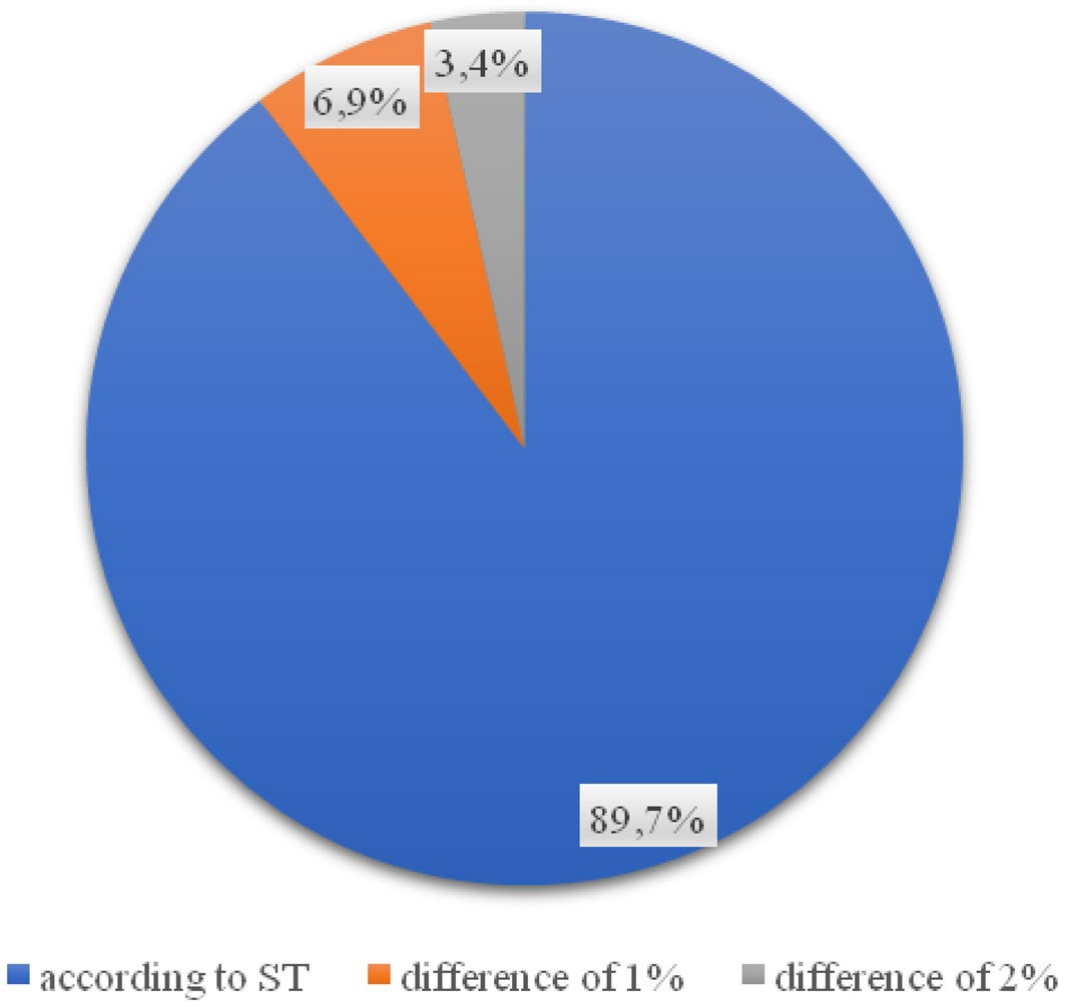
Percentage of the compaction index for the binding layer.

The percentage of compaction in the case of 3 samples was lower than the design requirements (Fig. 8), these are samples taken from Dobrzykowska, Polna and Raczkowizna streets.

The content of the free spaces in the binding layer was within the project's range of values in 19 cases. The remaining samples tested exceed the requirements of the technical specification by a maximum of 2%.

**Figure 9:**
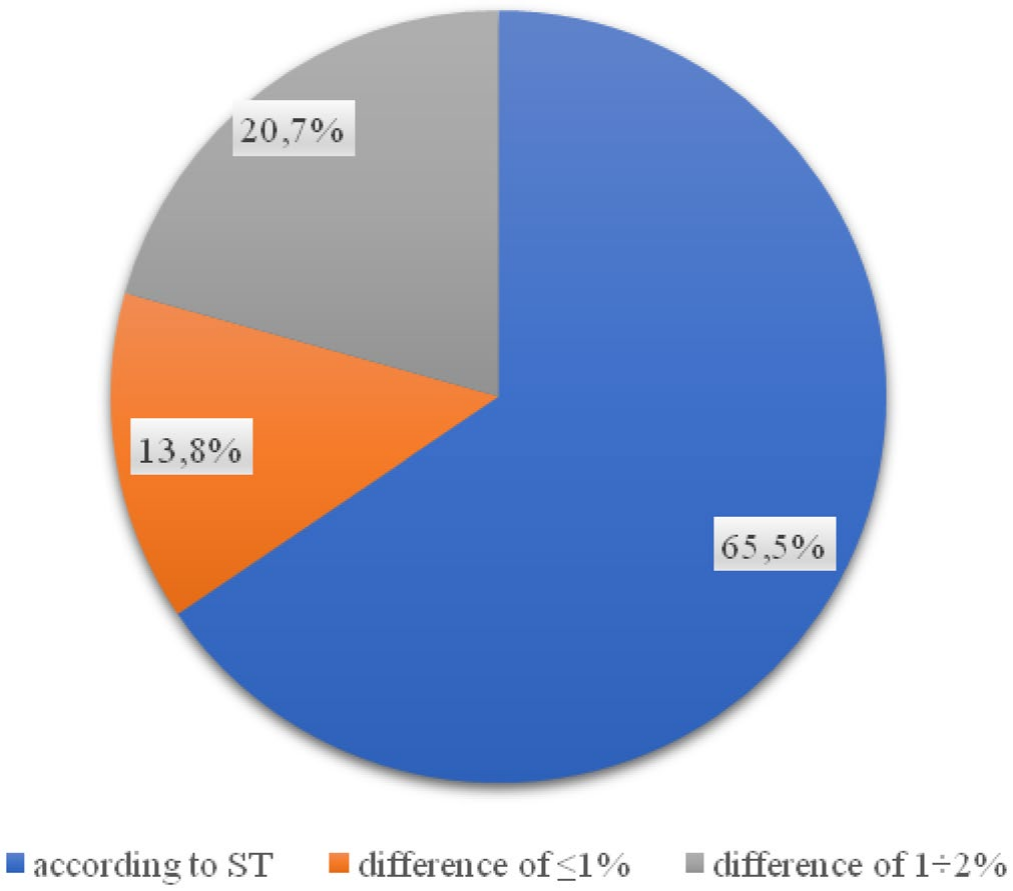
Percentage of values of free spaces in the binding layer.

**Figure 10:**
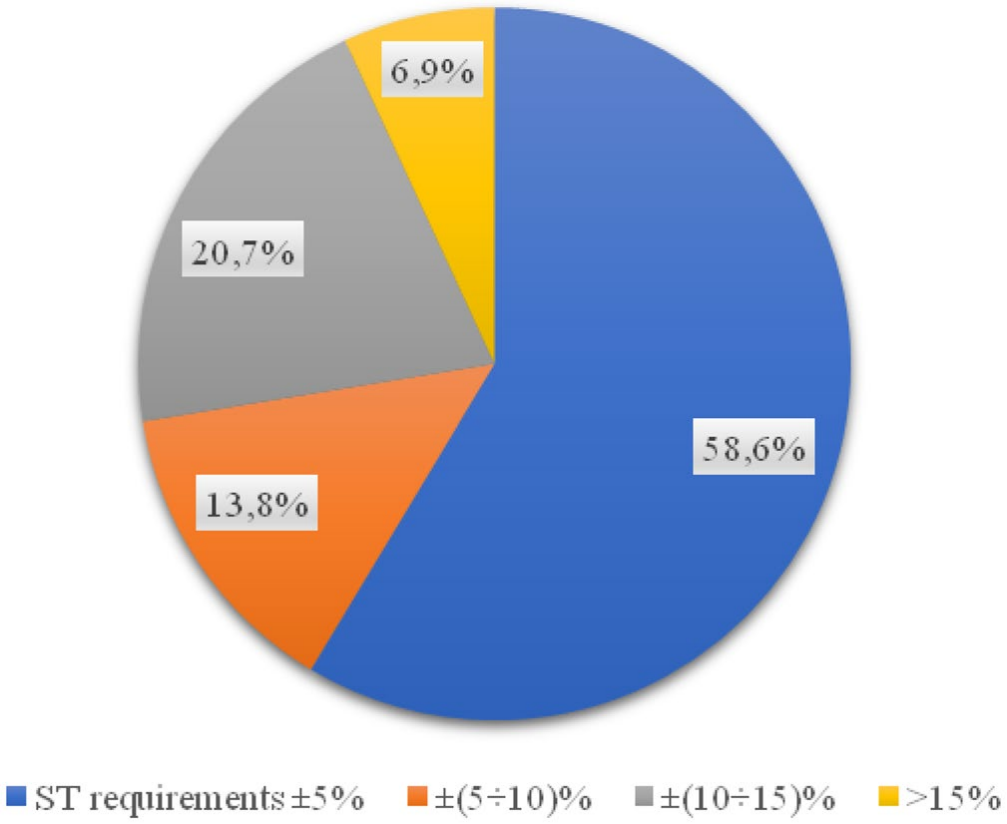
Percentage of the thickness of the wearing course in samples.

When analysing the wear layer, 21 out of 29 all tested samples met the requirements of the relevant technical specification (Fig. 9). Almost 28% of the surfaces were made in a manner inconsistent with the design documentation (Fig. 10). The wearing course was statistically characterized by the highest quality of pavement on individual streets. Differences in layer thickness compared to the assumptions of the technical specification ranged from 1 mm per street. Polna and Grabówka up to 7 mm on Otolińska and Zielona streets. In relation to the remaining layers, deviations from the expected value are the smallest.

**Figure 11:**
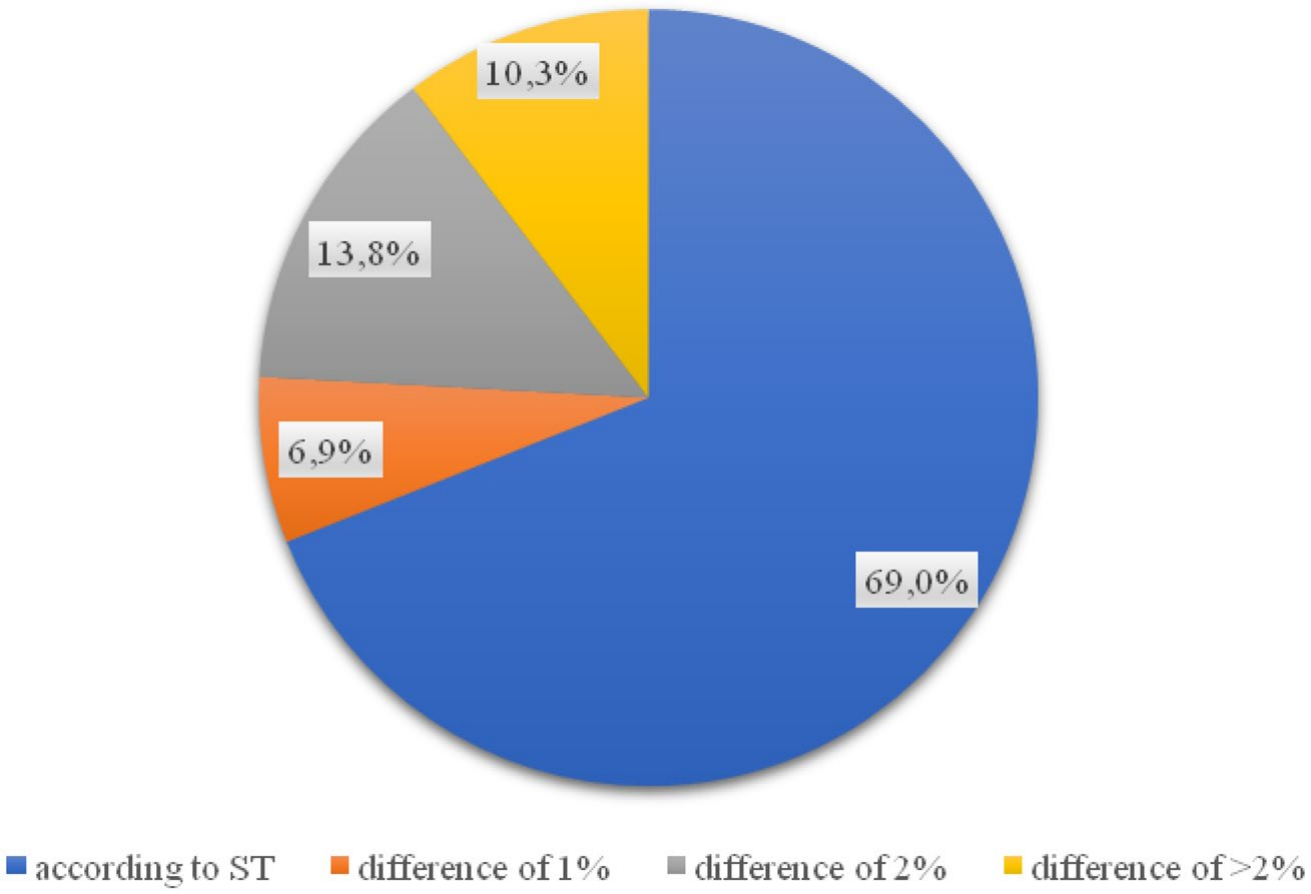
Percentage of the compaction index for the wearing course.

The compaction index study shows that as much as 31% of all surface samples taken were made without following the design guidelines (Fig. 11).

**Figure 12:**
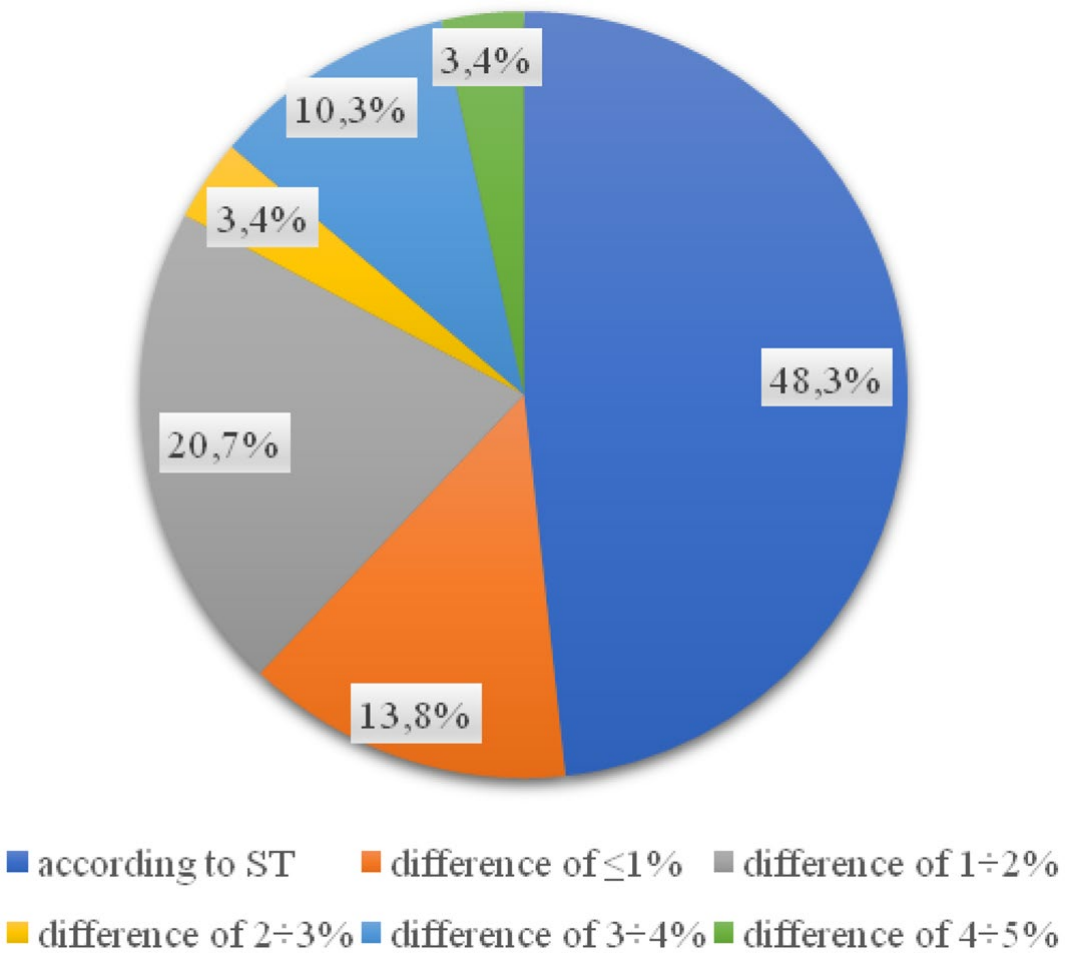
Percentage of free spaces in the surface course.

More than half of the results of free space tests in the layer do not fit within the strictly defined by the documentation range (Fig. 12). At the same time, for one of the core samples taken on Kolejowa Street, a result exceeding the limit value was obtained by more than 4%.

On all the streets examined, regardless of whether the project involved renovation, reconstruction or construction, the works included laying new layers of asphalt. In the case of renovations and reconstructions, the old layers were dismantled before new layers of asphalt were laid. In the case of new facilities, asphalt surfaces were laid on a properly prepared surface. The predicted variation in the thickness of individual layers was based on the traffic category assigned by the designer of each street, as shown in Table 2. The designed typical structures of the upper pavement layers, included in individual designs, were based on Table 1.

Deviations from technical specifications observed during testing may have various causes. The most common cause of implementation errors is inadequate supervision of road works, i.e. lack of systematic quality control, lack of qualified staff and selection of inappropriate equipment. Other reasons leading to deviations may include: improper surface preparation, incorrect composition of the asphalt mixture, as well as work carried out in inappropriate weather conditions, e.g. due to weather conditions during rainfall or low temperatures.

Comparing the three layers in terms of thickness—the largest number of execution errors occurred in the foundation layer, but the highest values of thickness deviations in relation to the technical specification requirements were recorded in the binding layer. The foundation layer showed the highest compliance with the technical specification in terms of the compaction index value, each sample met the design requirements. The wearing layer did not meet the imposed requirements in as many as 31% of the tested samples. The content of free spaces in the base and binding layers largely met the requirements of the technical specification, and if a value inconsistent with the specification was obtained, they were not greater than 2%. In the case of the wearing course, the maximum differences reached over 4%, and more than half of the samples did not meet the documentation assumptions.

## Summary

The assessment of the works carried out on selected road surfaces within the city of Płock (Poland) in terms of the requirements of the technical specification and the durability of the road infrastructure showed that implementation errors can translate directly into the durability of the surface. Tests of three road layers: the foundation layer, the binding layer and the wear layer were carried out assuming correctly prepared design documentation, assuming that the project was carried out in accordance with the requirements and guidelines contained in relevant documents and legal acts. The technical specification specifies detailed requirements constituting executive instructions for the implementation of a road investment. Failure to comply with the provisions of the technical specification may result in non-acceptance of works, increased costs, but also with an extension of the investment implementation time, often causing discomfort among users of reconstructed roads and adjacent buildings.

Invasive tests of the thickness of the substructure layer carried out on 11 streets of the city of Płock (Poland) showed that 58.6% of the analyzed road foundations met the requirements of the technical specification (ranging from 1 mm on Grabówka and Kostrogaj streets, to over 15 mm on Tysiąclecia and Zielona streets). 12 out of 29 samples taken do not meet the conditions of the technical documentation, and thus the investment in these places was not carried out in accordance with the project. The resulting value of the compaction index for each of the 29 samples is in line with the requirements, however, 20.7% of the analyzed samples do not comply with the technical specifications. For the binding layer, the results of the layer thickness tests showed that in 11 out of 29 samples the thickness of the binding layer does not comply with the guidelines contained in the technical design specification (from 2 mm on Zielona to as much as 23 on Kostrogaj Street). In the case of almost 28% of the surface, the wear layer was made in a manner inconsistent with the design documentation (from 1 mm on Polna and Grabówka to 7 mm on Otolińska and Zielona). Such discrepancies between the technical specification of the project and the execution of the road section may lead to the need for its re-repair, which is associated with the time of renovation.

Re-repairs may be associated with the need to remove the newly created surface and build materials with a composition that meets the assumptions adopted at the design stage, make layers of appropriate thickness or the right compaction index. Acceptance and use of a surface with manufacturing errors may result in loss of performance before the end of the design service life. This means that it may be necessary to carry out costly repairs earlier than submitted in the project.

Each investment requires an individual approach to implementation. If the deviations from the technical specifications are insignificant, the investor may allow the surface for use. In the event of major construction errors that may directly affect the safety of users by, for example, deep cracks, corrugations or ruts, it is necessary to remove layers that have not achieved the required quality characteristics. The removed layers can be used in recycled mineral and asphalt mixtures, which will reduce the carbon footprint of the material removed from the place of original installation. Making a decision to adopt surfaces with construction errors requires a broad analysis of economic, social and qualitative factors.

To improve the performance of asphalt pavement layers, close cooperation between contractors and the supervising inspector is recommended, along with regular inspections and quality control. Regular training of workers on new technologies and the use of modern equipment, taking into account prevailing weather conditions, can also contribute to improving the quality of pavement execution.

Both new and existing roads should be monitored to prevent the propagation of scratches, unevenness, material losses or ruts. This has a direct impact on the safety and comfort of road users.

## Data Availability

All data generated or analysed during this study are included in this published article.
